# Reply to letter to the editor in response to: “Carbon monoxide to ‘simulate altitude’”

**DOI:** 10.1113/EP093872

**Published:** 2026-06-01

**Authors:** Simone Villanova, Simone Porcelli, Lena Ekström, Daniele A. Cardinale

**Affiliations:** ^1^ Department of Movement, Human and Health Sciences University of Rome ‘Foro Italico’ Rome Italy; ^2^ Department of Molecular Medicine University of Pavia Pavia Italy; ^3^ Department of Laboratory Medicine, Division of Clinical Pharmacology Karolinska Institutet Stockholm Sweden; ^4^ Department of Physiology and Pharmacology Karolinska Institutet Stockholm Sweden

**Keywords:** endurance performance, erythropoiesis, haemoglobin mass, hypoxia, mitochondria

1

We thank Faiss and Brocherie (2026) for their comments on our recent paper (Villanova et al., 2026). We take this opportunity to clarify our position and the interpretation of our findings.

The study, along with the recent work published by our group (Urianstad et al., 2024), aimed to investigate the effect of carbon monoxide (CO) at the physiological level rather than to promote or incentivize the use of CO to enhance endurance performance. Our data show that chronic low‐dose CO exposure increased haemoglobin mass, red blood cell volume and blood volume. Importantly, these results did not translate into improvements in submaximal and maximal performance metrics investigated in the conditions tested. These findings do not support the use of CO exposure as a performance‐enhancing strategy.

Thus, our findings should not be interpreted as endorsing the use of CO in practice, which is prohibited for non‐diagnostic use under the current World Anti‐Doping Agency Prohibited List.

Additionally, our study evaluated standard haematological markers used to interpret changes in haemoglobin mass and included in the Athlete Biological Passport (ABP). Our findings showed no effects of low‐dose CO exposure on these variables compared with regular sea‐level training, except for a minor decline in immature reticulocyte fraction. However, these results were based on pre–post comparisons and did not replicate the longitudinal Bayesian framework of the ABP. Therefore, our data cannot be used to determine whether this intervention would be detected within the ABP framework and, as suggested by Faiss and Brocherie (2026), repeated CO exposure could be identified as an atypical passport finding within the ABP when evaluated using detailed longitudinal profiles.

Finally, we acknowledge that CO is a toxic gas and that any exposure carries risk. However, scientific exploration of these mechanisms, even when later deemed prohibited in sport, remains essential for understanding both performance biology and anti‐doping detection strategies.

Thus, we thank Drs Faiss and Brocherie for the opportunity to clarify these points and for the attention given to our study.

## AUTHOR CONTRIBUTIONS

All authors contributed to study conception and design. approved the final version, and agree to be accountable for the work. All authors have read and approved the final version of this manuscript and agree to be accountable for all aspects of the work in ensuring that questions related to the accuracy or integrity of any part of the work are appropriately investigated and resolved. All persons designated as authors qualify for authorship, and all those who qualify for authorship are listed.

## CONFLICT OF INTEREST

The authors declare no conflicts of interest.

## FUNDING INFORMATION

None.
